# The cancer preventative agent resveratrol is converted to the anticancer agent piceatannol by the cytochrome P450 enzyme CYP1B1

**DOI:** 10.1038/sj.bjc.6600197

**Published:** 2002-03-04

**Authors:** G A Potter, L H Patterson, E Wanogho, P J Perry, P C Butler, T Ijaz, K C Ruparelia, J H Lamb, P B Farmer, L A Stanley, M D Burke

**Affiliations:** Cancer Drug Discovery Group, School of Pharmacy, De Montfort University, The Gateway, Leicester LE1 9BH, UK; MRC Toxicology Unit, Hodgkin Building, University of Leicester, Leicester LE1 9HN, UK

**Keywords:** chemoprevention, resveratrol, piceatannol, cytochrome P450, CYP1B1

## Abstract

Resveratrol is a cancer preventative agent that is found in red wine. Piceatannol is a closely related stilbene that has antileukaemic activity and is also a tyrosine kinase inhibitor. Piceatannol differs from resveratrol by having an additional aromatic hydroxy group. The enzyme CYP1B1 is overexpressed in a wide variety of human tumours and catalyses aromatic hydroxylation reactions. We report here that the cancer preventative agent resveratrol undergoes metabolism by the cytochrome P450 enzyme CYP1B1 to give a metabolite which has been identified as the known antileukaemic agent piceatannol. The metabolite was identified by high performance liquid chromatography analysis using fluorescence detection and the identity of the metabolite was further confirmed by derivatisation followed by gas chromatography–mass spectrometry studies using authentic piceatannol for comparison. This observation provides a novel explanation for the cancer preventative properties of resveratrol. It demonstrates that a natural dietary cancer preventative agent can be converted to a compound with known anticancer activity by an enzyme that is found in human tumours. Importantly this result gives insight into the functional role of CYP1B1 and provides evidence for the concept that CYP1B1 in tumours may be functioning as a growth suppressor enzyme.

*British Journal of Cancer* (2002) **86**, 774–778. DOI: 10.1038/sj/bjc/6600197 www.bjcancer.com

© 2002 Cancer Research UK

## 

Resveratrol is a natural product found in red wine, and has been shown to have cancer preventative properties (Jang *et al*, 1997; Jang and Pezzuto, 1999). Resveratrol may be classified either as a polyphenol or as a phytoestrogen, and has the stilbene core structure. Piceatannol is a closely related phytoestrogen that also has the stilbene structure (Figure 1A

**Figure 1:**
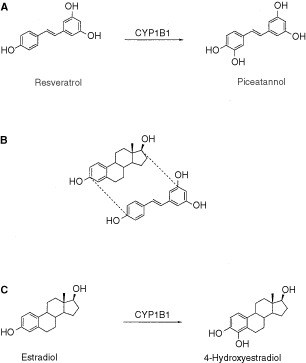
Molecular structures of resveratrol, piceatannol, estradiol, and 4-hydroxyestradiol. (**A**) Shows the conversion of resveratrol to piceatannol catalysed by CYP1B1. (**B**) Mapping of the phytoestrogen resveratrol onto the steroid framework of estradiol. (**C**) Shows the CYP1B1 catalysed aromatic hydroxylation of estradiol to 4-hydroxyestradiol.

). Piceatannol has known anticancer properties and was identified as the antileukaemic principle obtained from a plant extract (Ferrigni *et al*, 1984). Piceatannol was later found to exhibit potent tyrosine kinase inhibitory activity (Geahlen and McLaughlin, 1989). Subsequent studies have shown that it inhibits a variety of tyrosine kinases involved in cell proliferation, including the MAP kinases (Fleming *et al*, 1995), the tyrosine kinase involved in tubulin phosphorylation (Peters *et al*, 1996), kinases that are involved in the phosphorylation of DNA transcription factors (Su and David, 2000), and various other tyrosine kinases which are overexpressed in different types of cancer (Thakkar *et al*, 1993). The only difference between the stilbenes resveratrol and piceatannol is the presence of an extra hydroxy group in one of the aromatic rings of piceatannol.

The enzyme CYP1B1 belongs to the cytochrome P450 enzyme family (Spink *et al*, 1994; Sutter *et al*, 1994). Interestingly the CYP1B1 protein is highly expressed in human tumours (McKay *et al*, 1995; McFadyen *et al*, 1999). An immunohistochemical study has shown that the CYP1B1 protein is found in a wide variety of tumours of different organs such as brain, breast, colon, lung and ovary, but the protein was not detected in the corresponding normal tissues (Murray *et al*, 1997). Although the functional role of CYP1B1 is unknown, it is known to have aromatic hydroxylation activity, and catalyses the conversion of estradiol to 4-hydroxyestradiol (Spink *et al*, 1994; Hayes *et al*, 1996) (Figure 1C).

Resveratrol is classified as a phytoestrogen because of its similarity to the endogenous oestrogen estradiol. The molecular relationship between resveratrol and estradiol can be seen by mapping the molecular structure of resveratrol onto the estradiol framework as shown in Figure 1B. Because of this relationship we reasoned that resveratrol may also undergo aromatic hydroxylation by CYP1B1. Furthermore we rationalised that aromatic hydroxylation at the position corresponding to that of 4-hydroxyestradiol would generate the tyrosine kinase inhibitor piceatannol. We have used this type of mapping to design novel CYP1B1 activated tyrosine kinase inhibitor prodrugs for tumour selective cancer therapy using our concept of aromatic hydroxylation activation, and these prodrugs are based on the stilbene structure (Potter *et al*, 2001). We then realised the similarity in molecular structure of the anticancer prodrugs we had designed specifically for CYP1B1 to the molecular structure of certain natural products that have cancer preventative properties, and in particular the phytoestrogens such as resveratrol. This then led us to formulate a hypothesis for the functional role of CYP1B1 as a tumour suppressor enzyme or ‘rescue enzyme’ wherein CYP1B1 serves to activate certain non-toxic dietary components into growth inhibitory substances specifically within tumour cells containing the CYP1B1 enzyme. In this pilot study, we report here that resveratrol is indeed metabolised by CYP1B1 to generate the antileukaemic agent piceatannol.

## MATERIALS AND METHODS

Resveratrol and piceatannol were obtained from Sigma-Aldrich. Human lymphoblast expressed CYP1B1 microsomes were obtained from Gentest.

### CYP1B1 metabolism

Human lymphoblast expressed CYP1B1 microsomes (50 μl) were pre-incubated for 2 min at 37°C with 5 μl of stock 50 μM NADPH (final incubate conc.=1.25 μM). Pre-incubation volumes were made up to 198 μl with 0.1 M sodium/potassium phosphate buffer, pH 7.6. The reaction was started by the addition of 2 μl of 20 mM resveratrol in DMSO (final incubate conc.=200 μM). Final volumes were 200 μl. Incubations were carried out in air in a shaking water bath at 120 oscillations per min for 60 min at 37°C. The incubations were carried out under yellow light to avoid possible photochemical side-reactions. The reaction was stopped after 60 min incubation by adding 105 μl of an ice cold mixture of methanol (100 μl) and glacial acetic acid (5 μl) followed by placing on ice. The resulting mixture was centrifuged at 13 000 *g* for 10 min. One hundred microlitres of the supernatant was placed in a chromacol vial, and 60 μl aliquots analysed by high performance liquid chromatography (HPLC).

### HPLC analysis

HPLC was carried out using a Spectra-Physics SP8700 solvent delivery system, controlling a SP8750 pump, and a Gilson Model 231 sample injector with a 200 μl loop. Samples were injected onto a Kingsorb S5 C18 column (250×4.6 mm i.d.; Phenomenex) with a guard column housing a C18 cartridge (4×3 mm i.d.). Fluorescence detection was carried out with a Perkin Elmer LS 30 luminescence spectrometer, with excitation and emission wavelengths set at 345 and 405 nm respectively. Resveratrol, and its metabolites, were separated using an isocratic mobile phase composed of 25% acetonitrile and 75% 0.1 M formic acid (ammonium salt; Sigma) at a flow rate of 1.5 ml min^−1^, over 15 min. Under these conditions the following retention times were observed: resveratrol=12.45 min, piceatannol=6.91 min; M1=7.68 min, M2=6.91 min, M3=6.30 min.

### GC-MS analysis

Resveratrol and piceatannol trimethylsilyl (TMS) derivatives (Res-TMS and Pic-TMS, respectively) were prepared as follows. To a solution of the appropriate polyphenol (1 mg) in anhydrous DMF (50 μL) was added SYLON-BTZ (Supelco; BSA : TMSI : TMSCl, 3 : 3 : 2; 50 μL) and the mixture left for 30 min. Derivatisation of CYP1B1 metabolism samples were carried out as follows. An aqueous sample (9 ml) from the microsomal metabolism of resveratrol by CYP1B1 was extracted with ethyl acetate (3×5 ml). The combined extracts were dried (Na_2_SO_4_) and concentrated *in vacuo*. The residue was redissolved in anhydrous DMF (50 μL) under dry nitrogen, SYLON-BTZ (50 μL) added, and the mixture left for 1 h. The mixture was concentrated *in vacuo* and stored until use in the dark at −20°C. Gas chromatography–mass spectrometry analysis was performed on a VG 70SEQ mass spectrometer in electron ionisation (EI) mode (70 eV) coupled to a HP 5890 gas chromatograph with a PTE-5 30 m fused silica capillary column (Supelco). The injector temperature was 280°C. The derivatised samples of resveratrol and piceatannol (Res-TMS and Pic-TMS) were separated by GC under a variety of conditions. GC oven conditions of 80°C for 1 min→280°C@15°C min^−1^ gave retention times for Res-TMS (m/z 444) of 17 min 34 s and Pic-TMS (m/z 532) of 19 min 21 s. GC oven conditions of 80°C for 1 min→280°C@3°C min^−1^ gave retention times for Res-TMS (m/z 444) of 60 min 0 s and Pic-TMS (m/z 532) of 64 min 9 s.

## RESULTS AND DISCUSSION

**Figure 2:**
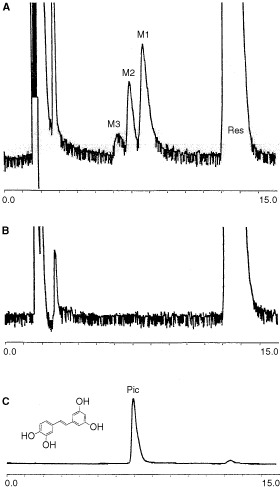
HPLC traces using fluorescence detection relating to the metabolism studies. (**A**) Shows the presence of three metabolites M1, M2, and M3 formed from the metabolism of resveratrol (Res) by CYP1B1 microsomes. (**B**) Shows the metabolism run without the NADPH cofactor. (**C**) Shows the elution profile of authentic piceatannol (Pic) containing a small amount of resveratrol.

**Figure 3:**
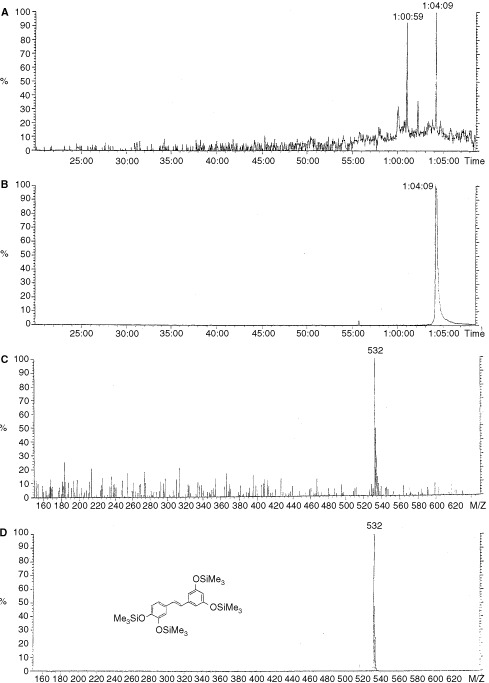
GC–MS studies on the derivatised metabolism sample. (**A**) Shows the GC–MS elution profile of the derivatised metabolism sample, displayed as a single ion chromatogram at m/z=532. (**B**) Shows the GC–MS elution profile, displayed as a single ion chromatogram at m/z-532, for derivatised authentic piceatannol (Pic-TMS; m/z=532). (**C**) Shows the mass spectrum of the metabolism sample peak that eluted at 64 min and 9 s. (**D**) Shows the mass spectrum of derivatised authentic piceatannol (Pic-TMS).

The metabolism of CYP1B1 was carried out using a microsomal preparation of the human CYP1B1 enzyme (Crespi *et al*, 1997). Using HPLC analysis with fluorescence detection we observed the formation of two major metabolites (M1 and M2) and one minor metabolite M3 (Figure 2A

). The major metabolite M2 has been identified as piceatannol (3,4,3′,5′-tetrahydroxystilbene). The other metabolites M1 and M3 have not been conclusively identified due to the unavailability of authentic standards. However we have deduced that M1 is probably 3,4,5,4′-tetrahydroxystilbene which results from 4-hydroxylation of the other aromatic ring, whilst M3 is possibly the corresponding dihydroxylated metabolite 3,4,5,3′,4′-pentahydroxystilbene. It is interesting to note here the striking similarity in molecular structure of these two putative metabolites to the structure of the highly potent anticancer agent Combretastatin A4 (3-hydroxy-4,3′,4′,5′-tetramethoxystilbene) (Dark *et al*, 1997). The other major metabolite M1 has been tentatively assigned here as 3,4,5,4′-tetrahydroxystilbene, and this stilbene has very recently been reported as an analogue of resveratrol that induces apoptosis in transformed cells, but not in their normal counterparts (Lu *et al*, 2001). Thus formation of the metabolite M1 could also make an important contribution to the chemopreventive activity of resveratrol. Metabolism studies were repeated with CYP1B1 obtained from a different source, using a CYP1B1 transfected *E. coli* enzyme preparation (Li *et al*, 2000), and this also gave the same metabolic profile (data not shown). Control experiments were carried out to validate these results by repeating the metabolism experiments, but systematically omitting a key component such as the substrate, the enzyme, or the essential cytochrome P450 reductase cofactor NADPH, and in each of these controls none of the metabolites were observed. The control experiment where the metabolism of resveratrol was carried out with the CYP1B1 microsomes, and only the NADPH cofactor was omitted, is shown in Figure 2B for comparison. The metabolite M2 was identified as piceatannol by HPLC coelution experiments with authentic piceatannol, and this gave the same retention time as M2. A spiking experiment was conducted where a small amount of authentic piceatannol was added to the metabolism sample following incubation, and HPLC analysis showed increased intensity of the M2 peak without any separation of this peak. In order to conclusively determine the identity of the metabolite M2 as piceatannol, mass spectrometry studies were undertaken, and a silylation derivatisation method was devised to enable sensitive GC–MS analysis. In this method exhaustive silylation of the metabolism sample was carried out so as to convert all hydroxlated components to their fully silylated derivatives. Authentic silylated materials were prepared for comparison and resveratrol was derivatised to its tris(trimethylsilyl) derivative (m/z=444) and piceatannol derivatised to its tetrakis(trimethylsilyl) derivative (m/z=532). The metabolism sample was then subjected to the exhaustive silylation procedure and analysed by GC–MS. Single ion monitoring at 532 was used to detect the GC elution profile of any components having a mass/charge ratio (m/z) of 532 corresponding to silylated piceatannol. In this way a peak was observed in the metabolism sample having a mass/charge ratio of 532 with an identical retention time as authentic silylated piceatannol. The GC conditions were varied widely to ensure that the peak was free from any extraneous signals and upon varying the GC conditions coelution with authentic silylated piceatannol was observed consistently. The most demanding GC conditions gave a retention time for silylated piceatannol of 1 h 4 min and 9 s, and this peak was clearly seen in the derivatised metabolism sample (Figure 3A,B

). This peak from the metabolism sample was then subjected to full mass spectral analysis, by operating in full scan mode, and this spectrum is shown in Figure 3C together with that of authentic silylated piceatannol in Figure 3D for comparison, which confirms its identity. Thus, using both HPLC separation with fluorescence detection and using GC with mass spectral detection, we have verified that the metabolism of resveratrol by CYP1B1 generates piceatannol as a major metabolite.

Resveratrol has been implicated as a chemopreventive agent in epidemiological studies, and a number of possible explanations for this activity have been proposed (Adhami *et al*, 2001; Mollerup *et al*, 2001; Wolter *et al*, 2001). In this pilot study we have demonstrated that this activity could possibly be due to the CYP1B1 mediated bioactivation of resveratrol. In the light of this discovery that CYP1B1 catalyses the conversion of a non-toxic dietary component into a compound with anticancer properties, we propose that the functional role of CYP1B1 is as a tumour suppressor enzyme, or ‘rescue enzyme’. Previously it has been suggested that CYP1B1 is present in tumours because it is the cause of tumours, since it has been shown to activate procarcinogens into carcinogens (Shimada *et al*, 1996; Kim *et al*, 1998). However this does not adequately explain the cause of tumours since there are many different mutagenic origins and oncogenic transformations that result in various forms of cancer, and yet CYP1B1 appears to be present in a wide variety of tumours irrespective of their oncogenic origin (Murray *et al*, 1997). In the context of our hypothesis that CYP1B1 is a tumour suppressor enzyme we need to make the pertinent point that, with respect to carcinogenesis, it does not matter if carcinogens are activated in cancer cells since they are already cancerous. Indeed tumour cells exposed to activated carcinogens may well die from the mutagenic damage, resulting in a cytotoxic effect (Shen *et al*, 1994). With respect to our hypothesis another relevant observation is that CYP1B1 is induced in skin cells on exposure to mutagenic UV light (Katiyar *et al*, 2000). Our hypothesis may also explain the cancer preventative properties of other phytoestrogens (Adlercreutz, 1995) such as flavones, especially as it has recently been found that some flavonoids are substrates for CYP1B1 (Doostdar *et al*, 2000).

In conclusion, the finding that resveratrol is converted into piceatannol by CYP1B1 has a number of important implications. Firstly, it provides a possible molecular mechanism for the cancer preventative properties of resveratrol. Secondly, it shows that a cancer preventative compound is converted into a compound with known anticancer activity by an enzyme that is found in human tumours. Thirdly, it provides supporting evidence for the hypothesis that CYP1B1 is a tumour suppressor ‘rescue’ enzyme, acting via natural prodrug bioactivation. Lastly, it validates the strategy of developing CYP1B1 activated prodrugs for cancer therapy which raises interesting opportunities for the treatment and prevention of cancer.
